# Single Shot High-Accuracy Diameter at Breast Height Measurement with Smartphone Embedded Sensors

**DOI:** 10.3390/s25165060

**Published:** 2025-08-14

**Authors:** Wang Xiang, Songlin Fei, Song Zhang

**Affiliations:** 1 School of Mechanical Engineering, Purdue University, West Lafayette, IN 47907, USA; xiang86@purdue.edu; 2 Department of Forestry and Natural Resources, Purdue University, West Lafayette, IN 47907, USA; sfei@purdue.edu

**Keywords:** LiDAR, RGB, diameter at breast height (DBH), single shot, smart phone

## Abstract

Tree diameter at breast height (DBH) is a fundamental metric in forest inventory and management. This paper presents a novel method for DBH estimation using the built-in light detection and ranging (LiDAR) and red, green and blue (RGB) sensors of an iPhone 13 Pro, aiming to improve measurement accuracy and field usability. A single snapshot of a tree, capturing both depth and RGB images, is used to reconstruct a 3D point cloud. The trunk orientation is estimated based on the point cloud to locate the breast height, enabling robust DBH estimation independent of the capture angle. The DBH is initially estimated by the geometrical relationship between trunk size on the image and the depth of the trunk. Finally, a pre-computed lookup table (LUT) is employed to improve the initial DBH estimates into accurate values. Experimental evaluation on 294 trees within a capture range of 0.25 m to 5 m demonstrates a mean absolute error of 0.53 cm and a root mean square error of 0.63 cm.

## 1. Introduction

Forests provide essential ecosystem services such as timber production and carbon storage. To manage forests well, accurate information at the individual tree level is needed. Diameter at breast height (DBH), measured at 1.4 m (4.5 feet) above the ground, is a key metric used to estimate tree size and timber volume [1]. Traditional tools like calipers and diameter tapes can measure DBH accurately, but they are time-consuming and labor-intensive, especially for large-scale forest inventories [2].

To improve efficiency, non-contact methods have been developed for DBH measurement. These include passive image-based photogrammetry and active laser scanning. Photogrammetry methods use 2D images to reconstruct tree shapes. Some studies applied calibrated cameras or smartphones with photogrammetry software to build 3D models for DBH estimation, achieving errors around 2–3 cm [3,4,5,6]. Other works used uncalibrated consumer cameras or GPS-based image alignment for tree mapping, reporting root-mean-square error (RMSE) between 1 and 3 cm [7,8]. Multi-view stereo approaches have also achieved sub-centimeter accuracy under controlled conditions [9]. While these methods show potential, they often require careful setup, multiple viewpoints, and complex processing, which limit their use in real-time applications.

Active laser scanning methods, such as airborne laser scanning (ALS) and terrestrial laser scanning (TLS), provide 3D point clouds by emitting laser pulses. ALS is effective for capturing large-scale forest structure, but its point clouds are often too sparse near the ground to estimate DBH accurately [10,11,12]. In contrast, TLS captures detailed point clouds from the ground level and has been widely used for precise DBH measurement, often achieving less than 5 cm RMSE [13,14,15,16,17]. Benchmarking studies have further confirmed the reliability of TLS in forestry applications [18]. However, TLS devices are expensive, require expert setup, and are not ideal for fast or widespread field deployment.

Mobile laser scanning (MLS) has emerged as a more flexible alternative to TLS. Systems mounted on handheld devices, backpacks, or UAVs offer improved mobility while maintaining reasonable accuracy. Studies comparing TLS and MLS have shown that MLS can achieve comparable or even better performance in some conditions, with up to 40% improvement in certain tasks [19,20,21]. However, professional-grade MLS systems remain costly and often require specialized software.

Recently, smartphones equipped with Light Detection and Ranging (LiDAR) sensors have gained attention as a low-cost, accessible MLS option. While early results are promising, challenges remain in achieving high accuracy and consistency with smartphone-based methods. Smartphone-based LiDAR systems and other MLS tools produce 3D point clouds of tree trunks, which can be used for DBH estimation [22,23]. Moreover, such smartphone-based LiDAR approaches remain limited in large-scale forest inventories due to their single-tree scanning workflow and manual operation. This process is inherently less efficient and scalable than automated or multi-tree scanning systems [23,24].

Common DBH estimation approaches include circle fitting, ellipse fitting, and cylinder fitting. Some studies used segmentation of the trunk followed by RANSAC-based cylinder fitting to determine orientation and DBH [25,26,27]. Others employed methods like the Gauss-Newton algorithm or Hough transform to fit circular cross-sections, achieving RMSE between 0.8 and 3 cm [28,29,30]. More advanced models that combined recursive fitting, multi-scan fusion, or functional cylinder modeling could reach higher accuracy under ideal conditions [31,32,33]. While these methods show strong performance, many rely on dense point clouds and precise single-point measurements from high-end sensors, limiting their use with sparse data captured by smartphones.

For real-time forest inventory, fast and simple DBH estimation methods are highly preferable. Several recent studies proposed mobile applications or lightweight models to speed up DBH measurement. These methods often use geometric rules, such as circle fitting or camera projection models, based on a single image or depth frame [2,34,35]. Reported errors range from 0.2 to 1.5 cm depending on tree size and viewing conditions. However, many of these approaches assume ideal conditions, such as perpendicular viewing angles or perfect circle-shaped trunks, and often ignore the tree’s growth direction. In addition, some methods rely on a small number of points, which may reduce robustness and accuracy in complex environments.

In this study, we propose a fast and accurate method for DBH measurement using a single RGB image and depth image captured by a smartphone with a built-in LiDAR sensor. Our method reconstructs a point cloud from LiDAR data and the RGB image, estimates the growth orientation of the tree trunk to locate the breast height. The DBH value is then estimated by the geometrical correlation between trunk size on the image and the depth values. A precomputed LUT is finally used to improve the initial estimated DBH value. Experimental results demonstrated the success of the proposed method, including evaluation on 294 trees achieving an MAE of 0.53 cm and an RMSE of 0.63 cm.

## 2. Materials and Methods

The proposed DBH estimation method involves reconstructing the 3D point cloud from a depth map and an RGB image captured by the iPhone sensors, segmenting captured data into the ground and tree trunk, estimating tree trunk orientation, and estimating initial DBH and further improving estimated DBH value using the pre-computed LUT. This section explain each step in details.

### 2.1. 3D Point Cloud Reconstruction

The world coordinate system is defined with its origin at the smartphone’s optical center, the $z^{w}$-axis pointing in the capture direction, and the $y^{w}$-axis pointing upward in portrait mode. We use the pinhole camera model [36] to relate 2D pixel coordinates (u,v) with 3D world coordinates $\left(x^{w},y^{w},z^{w}\right)$

(1) $$s{\left[u,v,1\right]}^{T}=P\cdot {\left[x^{w},y^{w},z^{w},1\right]}^{T},$$

where *s* is the depth at pixel (u,v), ^*T*^ denotes the matrix/vector transpose, P is the 3 row and 4 column projection matrix obtained from ARKit (iOS 16.4, Xcode 14.3, ARKit 6), and the symbol “·” is used to represent the dot product between vectors, or matrix multiplication, depending on the context. Since the depth value *s* is provided by the LiDAR sensor whose resolution is much lower than the RGB sensor, we address the resolution disparity between the high-resolution RGB image and the low-resolution LiDAR depth image by applying bilinear interpolation to estimate the depth at each RGB pixel. We then compute the corresponding 3D coordinates $\left(x^{w},y^{w},z^{w}\right)$ for each pixel using Equation (1), resulting in a point cloud that is spatially aligned with the RGB image.

### 2.2. Tree Trunk and Ground Segmentation

To segment ground and trunk pixels from the point cloud, we use the point prompt function of Segment Anything Model (SAM) [37] that requires seed pixels for segmentation. To automatically find seed pixels for SAM, we first compute the tangent vectors using the discrete Laplacian on the reconstructed 3D point cloud

(2) $$\begin{array}{c} T_{u}\left(u,v\right)={\left[\frac{\partial x^{w}}{\partial u},\frac{\partial y^{w}}{\partial u},\frac{\partial z^{w}}{\partial u}\right]}^{T}{|}_{\left(u,v\right)},\\ T_{v}\left(u,v\right)={\left[\frac{\partial x^{w}}{\partial v},\frac{\partial y^{w}}{\partial v},\frac{\partial z^{w}}{\partial v}\right]}^{T}{|}_{\left(u,v\right)}, \end{array}$$

where $T_{u}$ and $T_{v}$ denote the tangent vectors in the 3D space along *u*-direction and *v*-direction. We then compute the normal vector for each pixel as

(3) $$n\left(u,v\right)=\frac{T_{u}\times T_{v}}{|T_{u}\times T_{v}|}{|}_{\left(u,v\right)},$$

where n(u,v) denotes the unit normal vector at pixel (u,v) in the 3D space, and × denotes the cross product operator for two vectors.

We also obtain the gravity direction $n_{g}$ in the 3D space from iPhone’s ARKit. We assume the phone is held nearly vertical while the data is captured, and then the trunk pixels must satisfy the trunk condition

(4) $$|n_{g}\cdot n\left(u,v\right)|<\varepsilon_{t},$$

where $\varepsilon_{t}$ is a positive threshold. And the ground pixels satisfy the ground condition

(5) $$|n_{g}\cdot n\left(u,v\right)|>1-\varepsilon_{g},$$

where $\varepsilon_{g}$ is a positive threshold.

Finally, we define a square window at the image center with $M_{t}$ pixels in width and $M_{t}$ pixels in height. Among pixels within this window that satisfy the trunk condition in Equation (4), we choose the pixel with the smallest depth as trunk seed. For the ground seed, we compute the average depth of all pixels satisfying the ground condition in Equation (5) and select the pixel with depth closest to this average.

**Figure 1:**
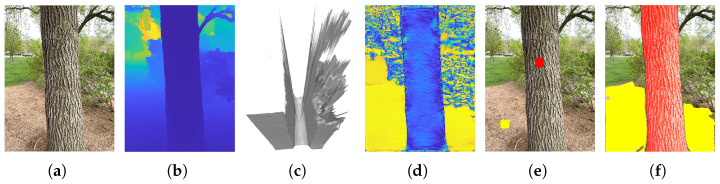
Tree trunk and ground segmentation. (**a**) Captured RGB image. (**b**) Captured depth image. (**c**) Reconstructed 3D point cloud. (**d**) $\left|n_{g}\cdot n\left(u,v\right)\right|$ value map. (**e**) Automatically determined seed pixels: the red dot indicates the trunk seed pixel, and the yellow dot indicates the ground seed pixel. (**f**) Segmentation result using SAM, with the tree trunk area shown in red and the ground area in yellow.

Figure 1 shows an example for the normal vector computation, where Figure 1a shows the captured high-resolution RGB image, and Figure 1b shows the the low-resolution depth image captured by LiDAR. Applying Equation (1) to the depth image will generate a low-resolution point cloud, which can be interpolated to create the full-resolution 3D point cloud for each RGB camera pixel, as shown in Figure 1c. Figure 1d shows the $\left|n_{g}\cdot n\left(u,v\right)\right|$ values, from which the ground pixels and trunk pixels are apparent. In Figure 1e, the automatically determined seed points are shown, with the trunk seed pixel marked in red and the ground seed pixel in yellow. Figure 1f displays the segmentation output from SAM, where the trunk area is highlighted in red and the ground area in yellow.

### 2.3. Growth Orientation and Breast Height Location Estimation

To locate breast height pixels on the tree trunk, we first estimate the tree’s growth orientation. We begin by fitting a plane function to the segmented ground points

(6) $$A_{g}x^{w}+B_{g}y^{w}+C_{g}z^{w}+D_{g}=0,$$

where $A_{g},B_{g},C_{g},D_{g}$ are the plane parameters from the Least Square fitting. We then compute the distance of each 3D point to the fitted plane as

(7) $$h_{⊥}\left(u,v\right)=\frac{\left|A_{g}x^{w}+B_{g}y^{w}+C_{g}z^{w}+D_{g}\right|}{\sqrt{A_{g}^{2}+B_{g}^{2}+C_{g}^{2}}},$$

where $h_{⊥}\left(u,v\right)$ denotes the distance from the point for pixel (u,v) to the ground plane.

Next, we estimate the growth orientation using a subset of trunk pixels with reliable geometric structure. Specifically, we select trunk pixels whose point-to-plane distances $h_{⊥}\left(u,v\right)$ lie within a predefined vertical range $\left[h_{1},h_{5}\right]$, where $h_{1}=1.0$ m and $h_{5}=1.8$ m. This interval is uniformly divided into four equal-height bands to form four stripes $S_{k}$, where k=1,…,4. Each stripe includes pixels satisfying $h_{k}<h_{⊥}\left(u,v\right)\leq h_{k+1}$, with a constant interval $\Delta h=h_{k+1}-h_{k}=0.2$ m.

Within each stripe on the segmented tree trunk, we find center pixels $\left(u^{*},v^{*}\right)$ along each image row and extract their corresponding 3D coordinates. These 3D points are then used to fit a line via least squares, estimating the local trunk orientation as

(8) $$\frac{x^{w}-x_{k,0}}{a_{k}}=\frac{y^{w}-y_{k,0}}{b_{k}}=\frac{z^{w}-z_{k,0}}{c_{k}},$$

where ${\left[x_{k,0},y_{k,0},z_{k,0}\right]}^{T}$ is a point on the fitted line, and $o_{k}={\left[a_{k},b_{k},c_{k}\right]}^{T}$ is the orientation vector for stripe $S_{k}$. The overall trunk orientation o is defined as the average of the four stripe orientations

(9) $$o={\left[a_{t},b_{t},c_{t}\right]}^{T}=\frac{1}{4}\sum_{k=1}^{4}o_{k}.$$

With the estimated trunk orientation o, we compute the height of each pixel (u,v) relative to the ground plane along the trunk orientation

(10) $$h\left(u,v\right)=\left|\frac{A_{g}x^{w}+B_{g}y^{w}+C_{g}z^{w}+D_{g}}{A_{g}a_{t}+B_{g}b_{t}+C_{g}c_{t}}\right|,$$

where $\left(x^{w},y^{w},z^{w}\right)$ is the associated 3D coordinates of the pixel (u,v) and the ground plane is obtained from Equation (6).

Among all center pixels $\left(u^{*},v^{*}\right)$, we then select those whose height $h(u^{*},v^{*})$ is closest to 1.4 m as the breast height center pixel

(11) $$\left(u_{0}^{*},v_{0}^{*}\right)=\arg\min_{\left(u^{*},v^{*}\right)}\left|h\left(u^{*},v^{*}\right)-1.4\right|,$$

where $\left(u_{0}^{*},v_{0}^{*}\right)$ denotes the 2D pixel coordinates of the center breast height pixel. And the associated 3D point is

(12) $$c_{0}={\left[x^{w}\left(u_{0}^{*},v_{0}^{*}\right),y^{w}\left(u_{0}^{*},v_{0}^{*}\right),z^{w}\left(u_{0}^{*},v_{0}^{*}\right)\right]}^{T},$$

where $c_{0}$ denotes the 3D center point at breast height for further computation.

**Figure 2:**
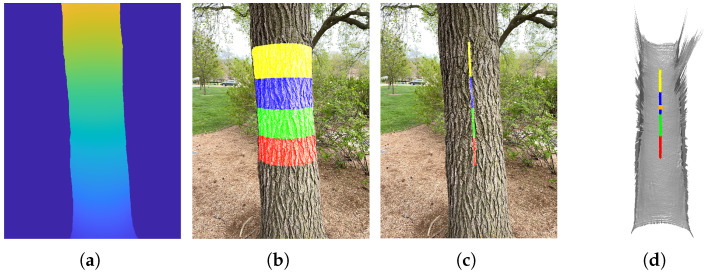
Basic concepts for tree’s growth orientation estimation. (**a**) Segmented trunk distance map $h_{⊥}\left(u,v\right)$. (**b**) Segmented stripes. (**c**) Center pixels $\left(u^{*},v^{*}\right)$ within those stripes. (**d**) Corresponding 3D points on the tree trunk surface, with the breast height center point highlighted in orange.

Figure 2a illustrates the typical vertical distance map $h_{⊥}\left(u,v\right)$ of a trunk. From this map, four horizontal stripes are obtained, as illustrated in Figure 2b. The center pixels $\left(u^{*},v^{*}\right)$ on each row within these stripes are highlighted in Figure 2c. Their corresponding 3D center points are shown in Figure 2d, where the breast height center point $c_{0}$ is marked in orange.

### 2.4. Initial DBH Estimation and Improvement

Given the estimated orientation o of the tree trunk and the fitted ground plane, we first identify pixels near breast height. A rigid body transformation is then applied to align the tree trunk orientation with the $y^{w}$-axis. Specifically, the 3D points are rotated so that the trunk’s orientation vector becomes parallel to the $y^{w}$-axis, and the chord direction becomes perpendicular to the $z^{w}$-axis. This alignment is achieved using

(13) $$\begin{array}{cc} \theta_{x}= & \arccos\frac{a_{t}}{\sqrt{a_{t}^{2}+b_{t}^{2}}},\\ \theta_{z}= & -\arcsin c_{t}, \end{array}$$

where $\theta_{x}$ is the $x^{w}$-axis angle (precession), $\theta_{z}$ is the $z^{w}$-axis angle (nutation), and ${\left[a_{t},b_{t},c_{t}\right]}^{T}=o$ is defined in Equation (9). The rotation matrix $R_{x}$ for $\theta_{x}$ and $R_{z}$ for $\theta_{z}$ can be expressed as

(14) $$R_{x}=\left[\begin{array}{ccc} \sin\theta_{x} & \cos\theta_{x} & 0\\ -\cos\theta_{x} & \sin\theta_{x} & 0\\ 0 & 0 & 1 \end{array}\right],\;R_{z}=\left[\begin{array}{ccc} 1 & 0 & 0\\ 0 & \cos\theta_{z} & \sin\theta_{z}\\ 0 & -\sin\theta_{z} & \cos\theta_{z} \end{array}\right],$$

and the composed rotation matrix R is

(15) $$R=R_{z}\cdot R_{x}.$$

The final transformed 3D points (x,y,z) can be computed as

(16) $${\left[x,y,z\right]}^{T}=\left[R,c_{0}-R\cdot c_{0}\right]\cdot {\left[x^{w},y^{w},z^{w},1\right]}^{T},$$

After transformation, circular geometry can be used to compute the tree diameter. Let ${\left[x_{i},y_{i},z_{i}\right]}^{T}$ be the associated 3D coordinates of a 3D point $c_{i}$. We estimate the DBH using the breast height pixel points

(17) $$\left\{c_{i}|1.39\;m<y_{k}<1.41\;m\right\},\;i=1,\ldots ,N_{\mathrm{BH}},$$

where $N_{\mathrm{BH}}$ is the number of breast height pixels (unit: pixel).

**Figure 3:**
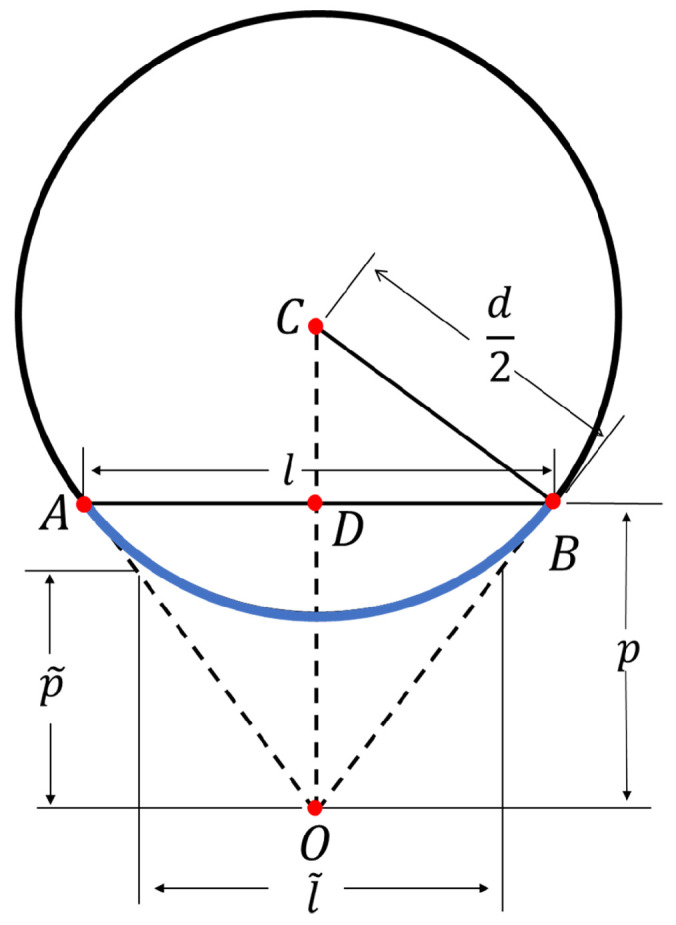
Diameter estimation based on circular geometry. Point *O* represents the camera’s optical center. Lines OA and OB are sight lines tangent to the circle, and the arc $\overset{⌢}{AB}$ represents the visible portion captured by the camera. The chord depth is given by $\bar{OD}=p$, and *d* denotes the diameter of the circle.

We apply the circular geometry for diameter measurement, as illustrated in Figure 3. Here *O* is the camera’s optical center, and *C* is the center of the circular cross-section of the tree trunk. Points *A* and *B* mark the endpoints of the visible arc $\overset{⌢}{AB}$ on the trunk, and *D* is the foot of the perpendicular from *O* to chord AB. The depth from *O* to AB is denoted by $p=\bar{OD}$, the chord length $l=\bar{AB}$ represents the straight-line distance between *A* and *B* in 3D space and *d* is the diameter of the circle.

The chord length *l* can be obtained using following equation

(18) $$l=p\frac{N_{\mathrm{BH}}}{f},$$

where *f* is the focal length obtained from iPhone’s ARKit (unit: pixel), and *p* is the chord depth (unit: cm).

Based on circular geometry, the diameter *d* of the trunk can be derived from the *l* and *p*

(19) $$d=\frac{l}{2p}\sqrt{l^{2}+4p^{2}}.$$

However, accurately determining the chord depth *p* from individual points *A* or *B* is challenging due to sparse LiDAR data points and sensor uncertainties. As a result, directly using *p* to compute the diameter *d* may lead to reduced accuracy. To address this, we approximate *p* using the average depth of all 3D points along the arc $\overset{⌢}{AB}$

(20) $$\tilde{p}=\frac{1}{N_{\mathrm{BH}}}\sum_{i=1}^{N_{\mathrm{BH}}}z_{i},$$

where $\tilde{p}$ is the average chord depth.

This approximation reduces noise impact but introduces systematic bias. The average depth $\tilde{p}$ is consistently lower than the true chord depth *p* and leads to an underestimation of the diameter. To address this, we compute the actual diameter *d* from the calculated $\tilde{p}$ and measured $N_{\mathrm{BH}}$ and *f*. Theoretically, the relationship between $\tilde{p},p,l,d$ can be expressed as

(21) $$\begin{array}{cc} \tilde{p} & =p+\frac{1}{2}\sqrt{d^{2}-l^{2}}-\frac{2}{l}\int_{0}^{l/2}\sqrt{\frac{d^{2}}{4}-x^{2}}\;dx,\\ \; & =p+\frac{1}{2}\sqrt{d^{2}-l^{2}}-\frac{d^{2}}{4l}\left(\arcsin\frac{l}{d}+\frac{l}{d}\sqrt{1-\frac{l^{2}}{d^{2}}}\right). \end{array}$$

Then the chord length *l* in Equation (19) becomes the underestimated chord length $\tilde{l}$

(22) $$\tilde{l}=l\frac{\tilde{p}}{p}=\tilde{p}\frac{N_{\mathrm{BH}}}{f}.$$

Substituting $\tilde{l}$ and $\tilde{p}$ into Equation (19), we obtain an estimate of the diameter

(23) $$\begin{array}{c} \tilde{d}=\frac{\tilde{l}}{2\tilde{p}}\sqrt{{\tilde{l}}^{2}+4{\tilde{p}}^{2}}, \end{array}$$

where $\tilde{d}$ is the initial estimate of the diameter and tends to underestimate the true value.

Finally, we improve the diameter value from the initial estimation. We have a system of five Equations (18), (19), (21)–(23) and five unknowns: $d,l,p,\tilde{d},\tilde{l}$. Given the measured values $N_{\mathrm{BH}}$, $\tilde{p}$ from sensors’ data and the known focal length *f* from iPhone’s ARKit, we can solve this system to get the true diameter *d* as the improved estimation.

**Figure 4:**
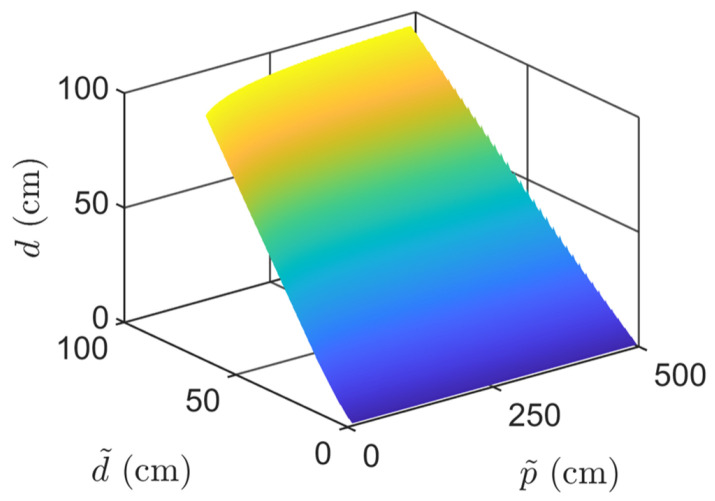
The relationship from $\left(\tilde{d},\tilde{p}\right)$ to *d*.

To avoid solving the nonlinear system at runtime, we pre-compute the mapping from $\left(\tilde{d},\tilde{p}\right)$ to the corresponding true values (d,p) and store the results in an LUT. Let $d_{\max}$ and $d_{\min}$ be the interested largest DBH and smallest DBH, $p_{\max}$ and $p_{\min}$ be the minimal chord depth and maximal chord depth. We then uniformly take $m_{1}$ samples of *d* in $\left[d_{\min},d_{\max}\right]$ and $n_{1}$ samples of *p* in $\left[p_{\min},p_{\max}\right]$ and compute the corresponding $\left(\tilde{d},\tilde{p}\right)$ using Equations (19), (21)–(23). This process gives a set of one-one correspondences between (d,p) and $\left(\tilde{d},\tilde{p}\right)$, from which the minimum and maximum values ${\tilde{d}}_{\min}$, ${\tilde{d}}_{\max}$, ${\tilde{p}}_{\min}$, ${\tilde{p}}_{\max}$ can be obtained. Figure 4 shows the relationship from $\left(\tilde{d},\tilde{p}\right)$ to *d*.

Then we create an $m_{2}$ rows $n_{2}$ columns LUT. We uniformly sample $m_{2}$ values of $\tilde{d}$ in $[{\tilde{d}}_{\min}$, ${\tilde{d}}_{\max}]$ and $n_{2}$ values of $\tilde{p}$ in $[{\tilde{p}}_{\min}$, ${\tilde{p}}_{\max}]$. Finally, we compute d for each sample using the previous one-one correspondences with barycentric interpolation [38]. Table 1 shows example values of the LUT, including the associated values d for selected combinations of $\left(\tilde{d},\tilde{p}\right)$.

In an actual measurement, once the average chord depth $\tilde{p}$ and the initial diameter $\tilde{d}$ are computed, the corresponding index (m,n) in the LUT can be calculated as,

(24) $$\begin{array}{cc} m & =\mathrm{ceil}(m_{2}\frac{\tilde{d}-{\tilde{d}}_{\min}}{{\tilde{d}}_{\max}-{\tilde{d}}_{\min}}),\\ n & =\mathrm{ceil}(n_{2}\frac{\tilde{p}-{\tilde{p}}_{\min}}{{\tilde{p}}_{\max}-{\tilde{p}}_{\min}}), \end{array}$$

where ceil() denotes the operator that determines the smallest integer greater than the input number. The diameter *d* value can then be found in the LUT using index (m,n). To further improve accuracy, four diameter values in the LUT are found using indices (m,n), (m−1,n), (m−1,n−1), and (m,n−1), and then bilinear interpolation is used to compute the final diameter value.

## 3. Results

We experimentally evaluate the performance of the proposed method. For the entire measurements, we used iPhone 13 Pro (Apple Inc., Cupertino, CA, USA) to capture all data. The RGB image resolution was set as 1440 pixels in width and 1920 pixels in height, and the LiDAR resolution was 192 pixels in width and 256 pixels in height. We used ARKit to extract camera intrinsic matrix and gravity direction vector. We set $\varepsilon_{g}=0.1$, $\varepsilon_{t}=0.3$ and $M_{t}=500$ pixels for all measurements in general. We set $d_{\max}=500$ cm, $d_{\min}=25$ cm considering the performance characteristics of the LiDAR sensor. The sampling parameters are chosen as $m_{1}=996$, $n_{1}=4996$, $m_{2}=500$, $n_{2}=1000$, which results in a resolution of 0.1 cm for true DBH estimation.

**Figure 5:**
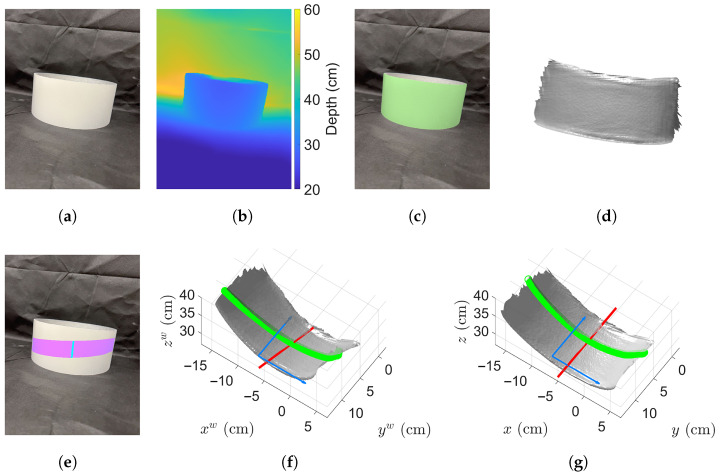
Example measurement of a testing cylinder with a diameter of 22 cm. (**a**) Raw RGB image. (**b**) Raw depth image. (**c**) Segmented cylinder. (**d**) Reconstructed 3D point cloud of the segmented cylinder. (**e**) Segmented stripe and the corresponding center pixels. (**f**) Initial cylinder orientation vector in red exhibits an angle between their respective axes where the green points denote the stripe arc points before transform. (**g**) Cylindrical surface after transformation where its orientation vector is parallel to the *y*-axis.

We first evaluated the proposed method by measuring an ideal cylinder (Model: MECCANIXITY Acrylic Pipe Rigid Round Tube ID 8.7", MECCANIXITY, Dragonmarts Co., Ltd., Kwai Fong, Hong Kong, China). The cylinder is made of transparent glasses, and was applied diffuse white spray paint by ourselves. Figure 5 shows the example of the cylinder being measured whose diameter is 22 cm. Figure 5a,b show the raw RGB and depth images. We then segment the cylinder from the background, as shown in Figure 5c. We also extracted the camera intrinsic matrix and reconstructed 3D point cloud of the segmented cylinder from the raw images, as shown in Figure 5d.

For an ideal cylinder, its orientation is uniquely defined, so a single stripe is sufficient for estimating its orientation. Figure 5e highlights the stripe with a height range of $h_{⊥}\in \left[4,8\right)$ cm in purple and the center pixels in cyan. Their corresponding 3D points of those center pixels are then used to estimate the orientation vector, which is $o={\left[-0.153,0.961,0.230\right]}^{T}$ in this case. With the estimated orientation vector, we transformed the reconstructed 3D point cloud so that the cylinder’s orientation aligns with the *y*-axis. Figure 5f shows the original 3D points, where the red line indicates the estimated orientation, and the green circular points represent the selected arc points used for diameter estimation via Equation (17). The blue arrows denote the directions of the $x^{w}$-axis and $y^{w}$-axis, respectively. It clearly shows that the cylinder’s orientation is not initially aligned with any coordinate axis. We perform the transformation defined in Equation (16), aligning the cylinder’s orientation with the $y^{w}$-axis. The result of this alignment is shown in Figure 5g, and now those green points denote the correctly selected arc points for diameter estimation.

For the segmented stripe, the total number of pixels $N_{\mathrm{BH}}=858$ pixels and the computed average chord depth is $\tilde{p}=29.84$ cm where the focal length is f=1451.99 pixels. Using these values, the chord length is calculated as $\tilde{l}=17.59$ cm, yielding an initial diameter estimate of $\tilde{d}=17.16$ cm. By referencing the pre-computed LUT with $\tilde{d}=17.16$ cm and $\tilde{p}=32.22$ cm, we have four pairs $\left(\tilde{d},\tilde{p}\right)=(17.10\mathrm{cm},32.00\mathrm{cm}$, (17.20cm,32.00cm), (17.10cm,32.50cm), and (17.20cm,32.50cm). The four corresponding d values are 22.48 cm, 22.32 cm, 21.96 cm and 21.82 cm. And the final estimated diameter is determined to be d=22.16 cm. In contrast, if the arc points were not transformed, the estimated diameter of the cylinder is d=21.42 cm.

**Figure 6:**
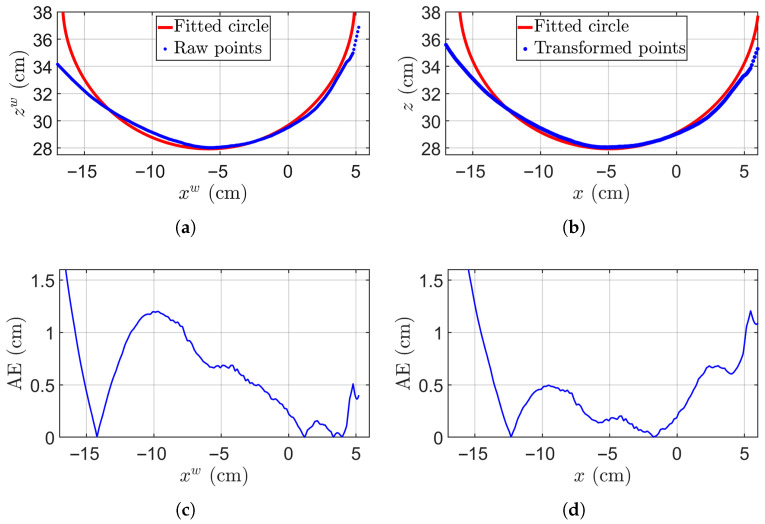
Arc points plot and absolute error distribution. (**a**) Arc points on raw surface and the corresponding computed circle with a diameter of 21.40 cm; (**b**) Transformed surface and the corresponding computed ideal circle with a diameter of 21.90 cm; (**c**) Absolute error for original arc points; (**d**) Absolute error for transformed arc points.

To further evaluate the accuracy, we fit the circle center using the estimated diameter *d*, and compute the absolute error (AE), mean absolute error (MAE), and the corresponding RMSE. For each measured 3D data point, we define the absolute error by taking the difference between the estimated radius (half of the diameter) and the actual distance from the point to the fitted center. In this example, the actual diameter is 22 cm. The selected arc points can are shown in Figure 6. The original arc points are represented in Figure 6a, while those on the transformed surface are highlighted in Figure 6b. The corresponding ideal circles are depicted by red curves with estimated diameters of 21.42 cm and 22.16 cm. The fitting circle centers are located at (−5.84cm,38.69cm) and (−4.96cm,38.97cm), respectively. For a visual representation of the accuracy of the circle fitted to the raw data, Figure 6c,d display AE for the two cases. The original surface yields MAE=0.63 cm and RMSE=0.78 cm. In contrast, the transformed surface results in an MAE of MAE=0.42 cm and RMSE=0.61 cm. This experiment clearly illustrates that the transformed arc points conform better to an ideal circle, given that the original arc points theoretically form an ellipse.

**Figure 7:**
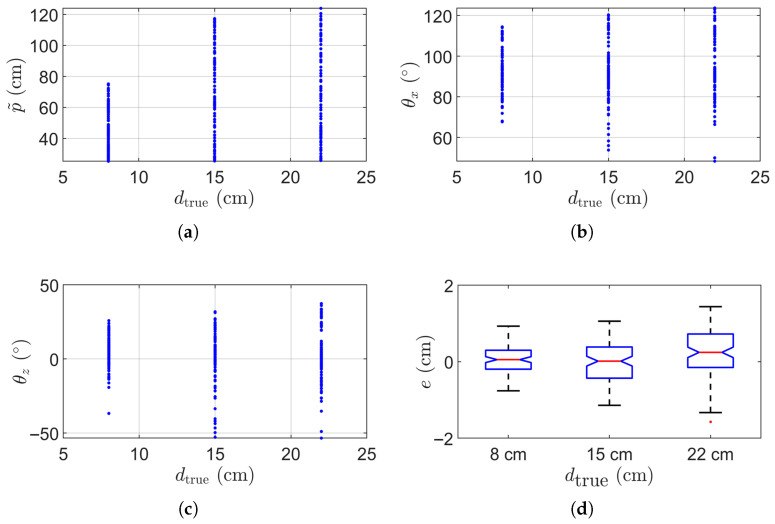
Statistical results of 300 cylinder measurements. (**a**) Estimated $\tilde{p}$ versus true diameter $d_{\mathrm{true}}$. (**b**) Estimated $\theta_{x}$ versus true diameter $d_{\mathrm{true}}$. (**c**) Estimated $\theta_{z}$ versus cylinder diameter $d_{\mathrm{true}}$. (**d**) Box plot of the diameter measurement error.

We proceeded to conduct a thorough evaluation of our proposed method, utilizing cylinders with three different diameters: $d_{\mathrm{true}}=$ 8 cm, 15 cm, and 22 cm. The corresponding estimated diameter error $e=d-d_{\mathrm{true}}$. Each cylinder was placed more than 20 cm from the camera and measured 100 times. Figure 7a shows the plot of $\tilde{p}$ versus $d_{\mathrm{true}}$, smaller cylinders are placed closer overall to ensure the cylinder to occupy at least 10% of the image. Figure 7b,c respectively shows the estimated $\theta_{x}$ and $\theta_{z}$ with respect to the diameter $d_{\mathrm{true}}$. As shown here, the cylinders were randomly oriented to test robustness. Figure 7d summarizes the distribution of errors across all 300 measurements. In all three cases, the diameter error remains within ±1 cm.

We then applied our proposed method to estimate the diameter of trees under real-world conditions. The tree measurements were sampled from the Horticulture Park, Purdue Campus and Martell Forest in West Lafayette, Indiana, United States. Trees of different species and diameters were selected, and major species in the study included the maples, oaks, walnuts, and pines. Total 294 individual trees were measured using a caliper (Model: Haglöf Sweden Mantax Blue 950 mm), and the caliper measured results were used as the ground truth. The tested tree diameters ranged from 15 cm to 95 cm, and the distance from the iPhone camera to each tree varied between 0.25 m and 5 m.

**Figure 8:**
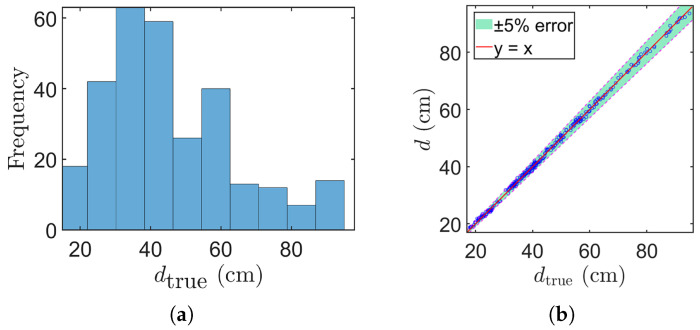
Tree diameter distribution and corresponding measurement results. (**a**) The DBH distribution of measured trees. (**b**) Comparison results between our method and caliper measurements. The dashed lines denote the ±5% relative error bounds and the blue circles represent measured data points.

Similar to the experiment conducted with standard cylinders, for each tree we used a single RGB–depth pair to compute the estimated diameter $d_{i}$, and compared it with the reference diameter $d_{\mathrm{true},i}$ measured by the caliper. A linear regression between *d* and $d_{\mathrm{true}}$ resulted in $d=0.997d_{\mathrm{true}}+0.233$, with a coefficient of determination $R^{2}=0.9988$, indicating excellent agreement. Figure 8a shows the distribution of tree diameters, and Figure 8b compares the estimated values with the ground truth. The overall MAE is approximately 0.53 cm, and the RMSE is around 0.63 cm, demonstrating the achieved high accuracy by our proposed method.

**Figure 9:**
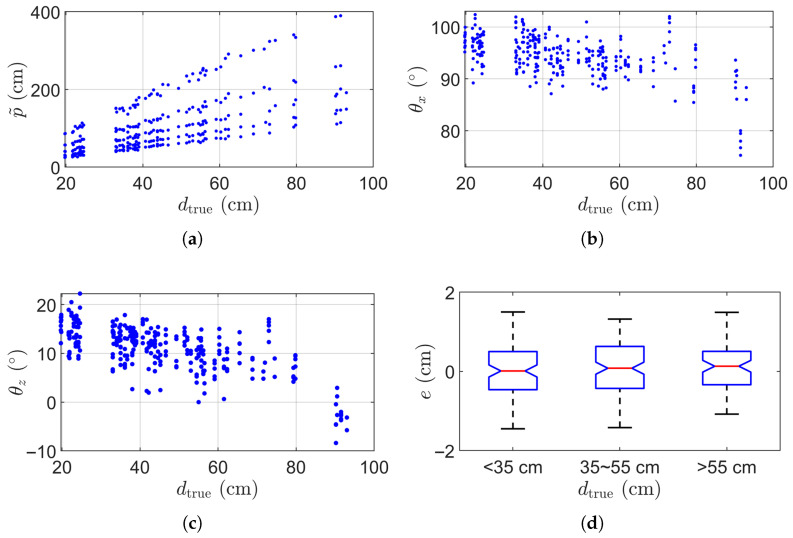
Statistical results of 294 tree measurements. (**a**) Estimated $\tilde{p}$ versus true diameter $d_{\mathrm{true}}$. (**b**) Estimated $\theta_{x}$ versus true diameter $d_{\mathrm{true}}$. (**c**) Estimated $\theta_{z}$ versus true diameter $d_{\mathrm{true}}$. (**d**) Box plot of the diameter measurement error.

Figure 9 presents statistical analysis of the cylinder measurement results across varying conditions. We group the tree into three sets based on its ground-truth DBH: <35 cm, 35~55 cm, and >55 cm. Figure 9a–c respectively shows the average depth $\tilde{p}$, the estimated $\theta_{x}$, the estimated $\theta_{z}$ with respect to the true DBH $d_{\mathrm{true}}$. Figure 9d summarizes the distribution of errors across all 294 measurements. In all three cases, the diameter error remains within ±1.5 cm.

**Figure 10:**
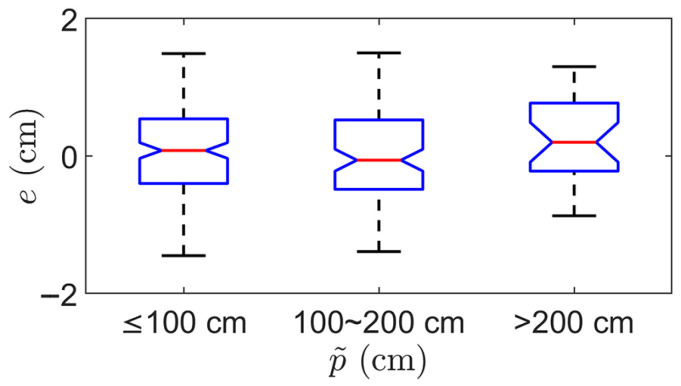
Grouped boxplot of DBH measurement error e for three ranges of average chord depth $\tilde{p}$. The three bins correspond to $\tilde{p}\leq 100$ cm, $100<\tilde{p}\leq 200$ cm, $\tilde{p}>200$ cm.

We further analyzed the relationship between the DBH measurement error e and the average chord depth $\tilde{p}$ using the tree dataset. The MAE is 0.52 cm, 0.55 cm, and 0.53 cm for $\tilde{p}\leq 100$ cm, $100<\tilde{p}\leq 200$ cm, $\tilde{p}>200$ cm, respectively. The corresponding RMSE values are 0.62 cm, 0.64 cm, 0.65 cm. These results are illustrated in Figure 10, which supports that the measurement error increases slightly with distance but remains within a sub-centimeter level across the full operational range.

These results confirm that the proposed method achieves high measurement accuracy for real trees using a single RGB image and a single depth image captured in one shot with an iPhone 13 Pro. Moreover, the accuracy remains consistent and is not influenced by the capture distance or the tree’s diameter.

## 4. Discussion

This study proposed a novel method to accurately measure tree DBH that only requires a single snapshot from a smartphone equipped with LiDAR technology. The proposed method has the following advantages

- *High Accuracy.* The proposed method achieved high accuracy through rigorous mathematical formation, and improved computational efficiency through approximation coupled with a pre-computed LUT. The method developed for the integrated sensor [2] estimates DBH by using the closest point on the circle at trunk cross-section to the camera center to determine the chord depth, and compute the diameter based on the circle geometry, achieving a best-case RMSE of 1.02 cm. The method designed for ARTreeWatch (Android Studio 4.0) [34] leverages motion tracking through visual-inertial odometry, along with feature and plane detection, followed by circle fitting for DBH estimation, resulting in a best-case RMSE of 1.04 cm. In comparison, our method achieves a lower RMSE of 0.63 cm, representing a clear improvement in accuracy.
- *High Efficiency.* Our proposed methodology significantly improves the efficiency of measuring DBH in forest settings. It takes approximately 20 s to perform each measurement with a caliper, whilst our smartphone-based approach requires less than one second. Our method is even more beneficial to measure large trees since it can be challenging to use a caliper to directly measure those large trees, or requires collaborative effort if a tape is used. The mobility offered by a smartphone, coupled with immediate data processing and storage capabilities, streamlines the entire measurement process.
- *High Flexibility.* Unlike those traditional methods that often require the image plane to align parallel to the tangential plane of the tree trunk at breast height to ensure accuracy, our approach relaxed such constraints by incorporating tree trunk orientation estimation and point cloud re-projection techniques prior to DBH estimation, thereby increasing flexibility of the data capture process.

**Figure 11:**
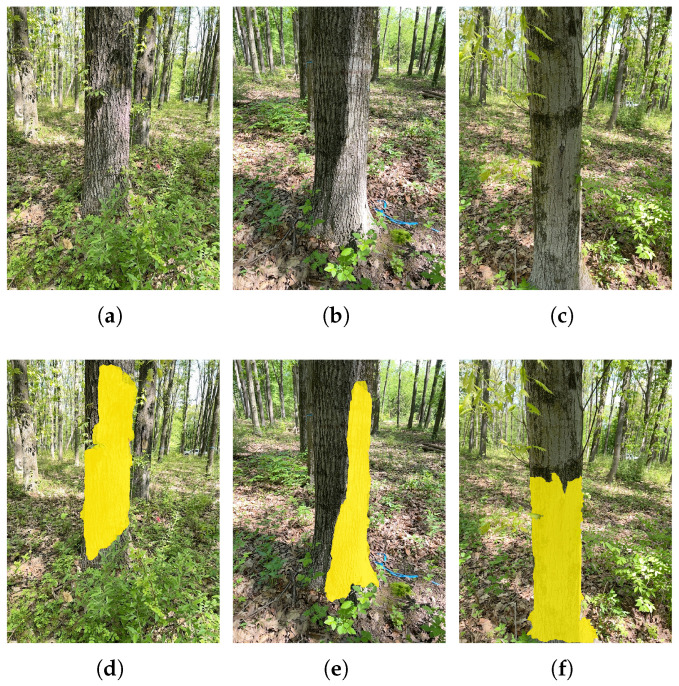
Representative examples of segmentation failures. (**a**) Example of the trunk partially obscured by understory vegetation and leaves. (**b**) Example of uneven illumination with a portion of the trunk appearing excessively dark. (**c**) Example of local variations in appearance due to intricate bark textures. (**d**–**f**) Corresponding segmented result of the example image above with yellow highlighting the segmented trunk area.

Yes, the proposed method is not trouble free. It has the following major challenges or limitations:

- *Depth range limit.* The limited depth range of the iPhone LiDAR (Apple Inc., Cupertino, CA, USA) sensor (i.e., 0.25~5 m) poses challenges if the tree is too small or too far away. Moreover, this proposed method assumes cylindrical tree trunk, the single snapshot method may not give an accurate DBH estimation if the tree trunk does not satisfy this condition.
- *Non-cylindrical trunk.* The proposed method assumed that the tree trunk is cylindrical, yet in natural forest, tree trunks exhibit deviations such as tapering, fluting, or leaning. Despite this, our forest measurement result is encouraging considering that we did not select trees whose trunks are close to be cylindrical but rather captured all trees within the sampled area. In practice, measurement from different perspectives could be taken to further improve DBH measurement accuracy.
- *Segmentation challenge.* Our proposed method assumed that the tree trunk has been recognized and segmented properly. However, tree trunk segmentation is extremely challenging. We found that SAM often fails if the tree trunk is not clean or the background is complex. To automatically and robustly recognize tree trunk, it is probably necessary to train a new artificial intelligence model specifically for this purpose. The current algorithm requires precise segmentation of the tree trunk and ground areas. It fails if the algorithm cannot detect tree trunk accurately either because the tree trunk is occluded (e.g., vines and leaves) at the DBH location when the trunk boundary cannot be precisely located. It might be also challenging in scenarios where understory vegetation or complex terrain obscure clear delineation. The problem becomes more complicated when the ground area has dense vegetation where the “ground” could be incorrectly detected. Segmentation failures occurred at a rate of 2.65% across the evaluated dataset, primarily due to partial occlusion, suboptimal lighting conditions, and complex trunk textures. Figure 11 presents three representative examples. Figure 11a shows understory vegetation and leaves partially obscure the trunk could fail segmentation and measurement, and the segmented trunk is shown in the yellow area in Figure 11d. Figure 11b shows uneven illumination (i.e., a portion of the trunk appear excessively dark) resulting in segmentation failure, as shown in Figure 11d. And Figure 11c shows local variations in appearance due to intricate bark textures introduce could mislead the model and cause segmentation errors, as shown in Figure 11f.

## 5. Conclusions

We have presented an novel method to accurately estimate the DBH of tree captured by the LiDAR and RGB sensors that are embedded in iPhone 13 Pro. The proposed method only requires a single depth image and a single RGB image within a snapshot for DBH estimation. Our method achieved sub cm accuracy for ideal cylinder measurements, and approximately 0.53 cm MAE and 0.63 cm RMSE accuracy for 294 trees located 0.25 m to 5 m away from the phone. The accuracy, flexibility, and speed of our proposed technique could significantly simplify the tree DBH measurements, contributing to the ecological health and economic profitability of forest ecosystems.

## Figures and Tables

**Table 1:** Example of LUT values: each cell contains *d* computed from a pair of $\tilde{d}$ and $\tilde{p}$.

	$\tilde{p}$ (cm)	50	150	250	350
$\tilde{d}$ (cm)					
10		10.69	10.21	10.11	10.07
30		35.73	32.22	31.37	30.99
50		63.43	55.93	53.75	52.75
70		92.33	81.04	77.16	75.29

## Data Availability

The code files and data files for replicating the results are available at open source Zenodo server https://doi.org/10.5281/zenodo.10650629.

## Abbreviations

The following abbreviations are used in this manuscript:

DBH	Diameter at breast height
AE	Absolute error
MAE	Mean absolute error
RMSE	Root mean square error
LUT	Lookup table
LiDAR	Light Detection and Ranging
RGB	Red, green and blue
